# Vaccinating Veterans Experiencing Homelessness for COVID-19: Healthcare and Housing Service Providers’ Perspectives

**DOI:** 10.1007/s10900-022-01097-1

**Published:** 2022-06-07

**Authors:** Michelle D. Balut, June L. Gin, Nikola R. Alenkin, Aram Dobalian

**Affiliations:** 1 Veterans Emergency Management Evaluation Center, U.S. Department of Veterans Affairs, 16111 Plummer St. MS-152, 91343 North Hills, CA USA; 2 Division of Health Services Management and Policy, The Ohio State University College of Public Health, 250 Cunz Hall, 1841 Neil Ave, 43210 Columbus, OH USA

**Keywords:** COVID-19 vaccination, Healthcare delivery, Healthcare providers, Homeless persons, Social support

## Abstract

This study examines challenges experienced during COVID-19 vaccination efforts, facilitating factors that increased vaccination, and lessons learned from healthcare providers and housing program staff who delivered healthcare and services to Veterans experiencing homelessness during the SARS-CoV-2 pandemic. Qualitative, semi-structured interviews were conducted with seven transitional housing program staff in northern California, southern California, Florida, Iowa, Kentucky, Massachusetts, and New Jersey (January-April 2021) and six primary care providers serving Veterans experiencing homelessness, four from clinics in California and two from a clinic in North Dakota (July-August 2021). Interviews were transcribed and analyzed using a rapid analysis approach. COVID-19 vaccination rates were between 40 and 60% among Veterans who received care from the primary care providers and between 20 and 90% among Veterans who were enrolled in the transitional housing programs. Barriers that providers and housing staff encountered when getting Veterans vaccinated for COVID-19 included lack of eligibility, the vaccine appointment scheduling process, transportation and communication challenges, Veterans’ distrust in the government, vaccine mandates, and vaccine hesitancy among organization staff. Recommendations to increase COVID-19 vaccine uptake included making vaccination more convenient, using trusted sources such as homeless program staff or Veteran peers to provide educational information about the safety and efficacy of COVID-19 vaccines, and encouraging rather than mandating vaccination. These lessons will enable entities providing care to people experiencing homelessness to develop more effective policies and educational campaigns to improve vaccine acceptance and uptake among this vulnerable population.

## Introduction

Vaccination for COVID-19 is the most recommended public health measure for reducing the likelihood of severe illness or death from severe acute respiratory syndrome coronavirus 2 (SARS-CoV-2) and controlling the pandemic [1]. However, as of March 3, 2022, approximately 35% of the U.S. population remains unvaccinated [2]. Commonly cited reasons for vaccine refusal include concerns about side effects, skepticism over the safety or efficacy of the COVID-19 vaccines, distrust in the government, believing that vaccination is not needed due to taking other precautions, or doubt in the severity of COVID-19 [3, 4]. For individuals experiencing homelessness, these explanations are further compounded by lack of access to regular healthcare and transportation, competing priorities, and a higher incidence of mental and physical impairments [5–7] which may hinder vaccination.

Previous research has found that having multiple primary care visits and being enrolled in housing programs were significantly associated with COVID-19 vaccination uptake among unhoused Veterans, likely due to convenient access to care and having trusted relationships with service providers [8]. Further, studies have shown that healthcare professionals are consistently regarded as one of the most trusted sources of COVID-19 vaccine information for people experiencing homelessness and other Veteran groups [9–12]. Therefore, it is important to understand whether and how organizations serving homeless populations are encouraging COVID-19 vaccination. This study examines challenges experienced during COVID-19 vaccination efforts, facilitating factors that increased vaccination, and lessons learned from healthcare providers and housing program staff who delivered care and services to Veterans experiencing homelessness during the pandemic.

## Methods

The study population included individuals who were employed at either (a) a U.S. Department of Veterans Affairs’ (VA) Homeless Patient Aligned Care Team (HPACT) or (b) a non-profit organization that is funded by the VA’s Grant and Per Diem (GPD) program. HPACT is a multi-disciplinary care model with clinics located in VA medical centers (VAMCs), community-based outpatient clinics (CBOCs), and community resource and referral centers (CRRCs, which provide access to services that promote permanent housing, healthcare, career development and access to VA and non-VA benefits) across the country. HPACTs consist of primary care providers, nurses, social workers, and mental health counselors who provide medical care, case management, housing and social services, community referrals, and substance use and mental health treatment [13]. GPD organizations provide transitional housing and social services to Veterans experiencing homelessness for up to two years [14].

The study team conducted qualitative, semi-structured, sixty-minute telephone interviews with six HPACT providers (two HPACT leaders, two primary care providers, and two nurses) in California (CA, n = 4) and North Dakota (ND, n = 2), as well as seven GPD organization staff (four leaders and three case managers) in northern California (N.CA), southern California (S.CA), Florida (FL), Iowa (IA), Kentucky (KY), Massachusetts (MA), and New Jersey (NJ). Sites were recruited through a self-selected convenience sample and were chosen for their program size and geographic variability. Interviews with GPD staff occurred between January and April 2021 and interviews with HPACT providers occurred between July and August 2021. Study participants were asked about their experience providing care/services during COVID-19; vaccination rates among their population; measures taken to encourage COVID-19 vaccination; challenges faced in getting their Veterans vaccinated; Veterans’ attitudes regarding COVID-19 vaccination and the pandemic; and suggestions to increase vaccine uptake. Interviews were audio recorded, transcribed, and analyzed using the rapid analysis approach [8, 15–17]. Further details regarding the recruitment methods, interview guide, and data collection and analyses are available elsewhere [17].

## Results

The nine HPACT and GPD programs in this study varied in size and Veteran patient population. The HPACT program in CA is one of the largest in the country and consists of 13 teams located within two VAMCs and one CBOC and serves approximately 2,600 Veterans. The four CA HPACT providers interviewed represented three clinical teams from one VAMC and the CBOC and served a combined total of 1,052 Veteran patients. The HPACT in ND is one of the smallest and newest programs, is within a CRRC, and served 110 Veterans at the time of the interviews. The GPD programs were all male only facilities with a total of 152 Veteran clients. The largest program, NJ, served 62 Veterans at the time of the interview while the smallest program, IA, served five.

Three themes emerged from the qualitative interviews with HPACT providers and GPD program staff: (1) COVID-19 vaccination rates and processes; (2) challenges to vaccinating Veterans for COVID-19; (3) facilitators to COVID-19 vaccination and suggestions to increase COVID-19 vaccine uptake.

### Theme 1: COVID-19 Vaccination Rates and Processes

#### Vaccination Rates

Table 1 illustrates the number and percentage of Veterans vaccinated for COVID-19 by site as reported by GPD organization staff and HPACT providers on the date of their interviews. Vaccination rates among the GPD sites varied. The Iowa organization had only vaccinated 1 out of 5 Veterans by 2/16/2021 because most Veterans were not yet eligible. Around half of the Veterans at most GPD sites, including NJ (32–40%), KY (40%), N.CA (44%), MA (50%), and FL (60%), had been vaccinated for COVID-19 by early-February to mid-March 2021. The S.CA site had the highest vaccination rate (90% by 2/24/2021).

**Table 1:** Estimated COVID-19 vaccination rates in GPD and HPACT programs, by site

Site	Interview Date(s)	# Veterans in Program^1^	# Veterans Vaccinated^1,2^	% Veterans Vaccinated^1,2^
**GPD**				
FL	1/26/2021	25	15	60%
MA	2/1/2021	8	4	50%
KY	2/11/2021	15	6	40%
IA	2/16/2021	5	1	20%
NJ	2/19/2021	62	20–25	32–40%
S.CA	2/24/2021	10	9	90%
N.CA	3/16/2021	27	12	44%
**HPACT**				
CA^3^				
Team 1	7/27/2021	342	173	50.5%
Team 2	8/2/2021	317	159	50.1%
Team 3	8/20/21	393	173	44%
ND	8/23/2021 8/31/2021	110	-	~ 60%
^1^ as of interview date ^2^ one or more dose ^3^ # and % fully vaccinated (2 doses) pulled from local clinical dashboard 7/30/2021				

CA HPACT utilized a local clinical dashboard to track COVID-19 vaccination numbers in the 13 clinics. The dashboard included both vaccinations within the VA and data from the California Immunization Registry (CAIR) [18], which identified vaccination that occurred at non-VA providers. Based on the dashboard, study participants reported that 40–50% of their patients were fully vaccinated (received both doses) for COVID-19 as of 7/30/2021. However, one respondent noted that dashboard accuracy may be an issue as data from CAIR was irregularly imported and the registry did not include Veterans who were vaccinated outside of California.

ND HPACT did not have a local data dashboard but rather pulled VAMC-level vaccination rates from VHA Support Service Center’s (VSSC) COVID-19 Vaccine Tracker. Based on their internal clinic records, the study participants estimated that around 60% of their Veterans were vaccinated for COVID-19 as of 8/31/2021.

#### Vaccination Process

At the time of the GPD staff interviews in early 2021, Veteran residents of the sites in NJ, N.CA, S.CA, KY, and IA had to make appointments at their VAMC to receive a COVID-19 vaccine. GPD staff in NJ and N.CA worked with VA staff to schedule appointments for Veterans. The S.CA respondent noted that the VA was contacting Veterans to schedule appointments. Both the FL and MA GPD arranged for clinical teams from the VA and a local pharmacy, respectively, to come vaccinate Veterans onsite. At both sites, Veterans who were unable to get vaccinated onsite could make appointments at their VAMC.

HPACT providers cited that appointments were required for the first several months of the vaccine rollout; however, Veterans were able to walk-in and get vaccinated at the VA without appointments by summer 2021. CA providers at the VAMC noted that the COVID-19 vaccine could be administered near the HPACT clinic until May 2021, but later, Veterans had to walk to the main medical building to get vaccinated. Both appointment and walk-in processes required close coordination across the nursing, pharmacy, and scheduling teams. Veterans in ND could not be vaccinated at the HPACT clinic and typically had to travel to the main VAMC to receive a COVID-19 vaccine by appointment or walk-in. However, providers hosted two “vaccination blitzes” in the ND HPACT clinic in early February 2021 (see Facilitators to COVID-19 Vaccination for further detail).

### Theme 2: Challenges to Vaccinating Veterans for COVID-19

Respondents in HPACT and GPD programs cited organizational and individual-level challenges to getting Veterans vaccinated for COVID-19, see Table 2, column 1.

**Table 2:** – COVID-19 Vaccination: Challenges, Facilitators and Lessons Learned

Challenges to vaccination	Facilitators to vaccination and lessons learned
*Policy and organization-level*	
• Lack of availability/ineligibility	
• Appointment scheduling process	• Facilitate easy access to vaccines
• Transportation/distance to clinics	
• Poor communication between VA, GPD organizations, and Veterans	
*Veteran individual-level*	• Provide educational information
• Competing priorities	• Leverage trusted sources of information
• Distrust of government	• Encourage rather than mandate
• Vaccine mandates creating barriers	
*GPD staff individual-level*	
• Staff vaccine hesitancy	

#### Policy and Organizational-Level Challenges

GPD staff in IA and NJ expressed that lack of availability was initially a major barrier to rapidly vaccinating Veterans. The state of Iowa did not prioritize persons experiencing homelessness during the initial vaccine rollout and the respondent conveyed frustration at the lengthy wait time. In addition, most of the Veteran residents in IA were not eligible for VA healthcare, so they were unable to get the vaccine at the VA in early 2021 (Note: In March 2021, the SAVES LIVES ACT expanded COVID-19 vaccine eligibility to all Veterans and their spouses, regardless of VA healthcare enrollment status [19]). In addition, the FL GPD respondent expressed anger that staff were ineligible to get vaccinated when the VA vaccination team came to their site:

*“We’re healthcare providers. I’ve got 16 staff. So, far, none of them have been vaccinated… And I’ve had two staff test positive... I have written congressmen. I have tried to e-mail the [VAMC] director. I have tried to go to Washington and e-mail the VA there. And I’ve talked to the Health Department… and this is the story I get: You’re not VA employees, even though you contract with them, so they don’t care about you…They don’t care if we get [COVID-19] from the [Veterans]. They just want to make sure that we help them… So, that’s my big frustration, and that’s why I call them the forgotten healthcare providers.” (FL GPD)*

For CA HPACT clinicians, the appointment scheduling process was a major challenge before walk-in vaccination was available to Veterans. According to a HPACT respondent, neither HPACT nurses nor HPACT scheduling clerks were given permission to schedule vaccine appointments for patients; instead, a “return to clinic” order had to be issued by a primary care provider and then a scheduling clerk from a regular PACT clinic could schedule an appointment. This frustrated the vaccination efforts:

*“The clerk in regular PACT actually would have been able to schedule a Veteran into the vaccine clinic for an appointment themselves… our clerks were never given the permission for that. So in some ways our Veterans were not just treated as normal, they were actually worse off than Veterans in other clinics. So that was a pretty frustrating experience… There were definitely times when we were feeling left out during COVID.” (CA HPACT4)*

HPACT providers in CA and ND cited transportation barriers in the early days of the COVID-19 vaccine rollout. COVID-19 vaccines were initially only offered at the main VAMCs, so patients receiving care at a CBOC or CRRC had to travel to the main campus, which was inconvenient and intimidating for most HPACT Veterans. Additionally, the VA shuttle between clinics in CA was discontinued during the height of the pandemic to minimize exposure risk. ND providers also noted challenges getting the COVID-19 vaccines to rural CBOCs:

“*Well probably 50% of the Veterans we serve aren’t located in the [city] area, they’re located in highly rural areas, and getting the vaccines out to the remote clinics, and we even have some clinics that are located in what’s still considered ‘frontier’ areas… It was a huge logistical effort to have the vaccines there, the staff there to deliver the vaccines, to care for people post-vaccine, yeah, it was a big logistical effort to make sure that happened...” (ND HPACT2)*

Respondents in HPACT and GPD identified communication challenges regarding COVID-19 vaccine availability. One CA HPACT provider believed that the VAMC did not effectively announce to VA staff and Veteran patients when and where vaccines were available. A NJ GPD staff member also stated that the VAMC did not give sufficient advance notice of vaccine availability to get Veteran residents signed up and arrange transport to the VAMC. Further, miscommunication between CA HPACT providers and local GPDs about which COVID-19 tests were required for GPD program entry exacerbated workload challenges for HPACT providers.

#### Veterans’ Individual-Level Challenges

Providers in both the HPACT clinics and GPD organizations noted that Veterans themselves likely did not experience many logistical barriers to receiving a COVID-19 vaccine since there were so many resources available to them. However, they cited various personal challenges hindering vaccination. For example, N.CA and NJ GPD staff mentioned that many Veterans have jobs or other competing priorities that may inhibit vaccine uptake:

*“We have some who are like oh I’m gonna do it… I’ve just been busy with work. Or I’m gonna do it, I’ve just been depressed…. Or they’re going through the mental health stuff. Or they’re struggling with their addiction. So it’s not a priority for them. Because they’re distracted by these other things.” (N.CA GPD)*

Participants in both HPACT and GPD cited Veterans’ distrust and cynicism as a barrier to COVID-19 vaccination. One HPACT provider in CA noticed that patients who were skeptical of other vaccines were also refusing the COVID-19 vaccine and that many of his Veterans cited the Tuskegee experiments as their reasons for distrusting vaccination. Two respondents, one from the HPACT clinic in CA and another from a GPD in NJ, noted that the younger Veterans, particularly those from the Operation Enduring Freedom/Operation Iraqi Freedom (OEF/OIF) generation (who served in the U.S. wars in Afghanistan and/or Iraq that began in 2001 and 2003, respectively), were more distrustful of the government and the VA, and thus more resistant to COVID-19 vaccination.

HPACT providers and GPD staff had differing views on the topic of mandating the vaccine. Some CA HPACT providers thought a mandate would help facilitate vaccine uptake, believing that if GPD programs or prospective employers made vaccination mandatory, more Veterans would get vaccinated. However, one ND provider opined that a vaccine mandate would create a *“power struggle dynamic”* for Veterans, exacerbating their distrust of the vaccine and cause them to *“dig in”* deeper in their opposition to vaccination. Instead, they suggested that trusted sources should encourage vaccination:

*“What I think I have seen that’s been more effective is when there’s more privileges allowed to those who have been vaccinated… Our GPD provider here in [city] has not mandated the vaccine yet… in my opinion it’s easier to work with Veterans through their case manager, through their trusted health provider to educate them about getting vaccines, rather than trying to force it.” (ND HPACT2)*

At the time GPD staff were interviewed, vaccine mandates were not being considered a requirement for admission to transitional housing. Only the KY GPD stated that they considered making COVID-19 vaccination mandatory to program entry. Like ND HPACT2, the IA GPD respondent believed that mandating the vaccine for Veterans would create a barrier to accessing homeless services:

*“I have made the decision that… I will never make it mandatory for clients. That’s way too much of a barrier for our clients that already have a world full of barriers on their shoulders. And we are here to break down those and not set up more and requiring a vaccination… I’m not gonna put that barrier on our clients.” (IA GPD)*

#### GPD Staff Individual-Level Challenges

GPD providers in NJ and IA mentioned that COVID-19 vaccine hesitancy was a major challenge among their staff. The NJ participant said that 100% of their unvaccinated staff are African American and had expressed that they don’t trust the vaccine. The respondent worried that staff who decline vaccination may not be effective advocates to encourage Veteran vaccination. The IA respondent noted that two staff members would quit their jobs if the organization made COVID-19 vaccination mandatory for employment:

*“I actually had two staff members come talk to me separately and asked if I… was going to make the COVID-19 vaccine mandatory for all staff. And I had told them like I’m still doing my research, that hasn’t been determined yet. And both had said if it happened, I’ll be giving my resignation. And that they will not be getting the vaccine... It definitely caught me off guard.” (IA GPD)*

The N.CA and MA respondents both reported initial apprehension about getting vaccinated but, upon learning more about the vaccine, ultimately overcame their hesitancy to protect their families.

### Theme 3: Facilitators to COVID-19 Vaccination and Suggestions to Increase Uptake

Accessibility, educational information, and trust were the primary factors that both HPACT providers and GPD staff identified as influential in increasing Veterans’ uptake of the COVID-19 vaccines. See Table 2, column 2.

#### Improving Ease of Access

The ND HPACT was able to get a high proportion of their Veterans vaccinated by holding two “blitzes”- special vaccination days at the HPACT clinic where they provided free food and giveaways and health professionals were available to answer Veterans’ questions about the vaccine. The HPACT clinic is more convenient and less intimidating than the VAMC, they said, so Veterans likely felt more comfortable getting vaccinated there. Further, ND respondents believed that seeing their peers getting vaccinated may have encouraged Veterans to get the vaccine.

The GPD organizations in FL and MA facilitated uptake by having a VA and pharmacy team, respectively, come to their sites to administer the vaccine, making it easier and reducing both physical and perceived barriers for Veterans. In MA, having a team from the local pharmacy also improved access as even Veterans not eligible for VA healthcare could get the vaccine. Additionally, N.CA, NJ, and MA GPD respondents mentioned that the VA called both GPD staff and Veterans to let them know when the COVID-19 vaccine was available and helped them make appointments. All seven GPD sites provided transportation for Veterans to the VAMC if they wanted to get the COVID-19 vaccine.

HPACT and GPD providers agreed that lowering systemic barriers to accessing vaccines could improve uptake among Veterans experiencing homelessness, who already navigate a plethora of daily challenges. GPD staff in NJ and KY suggested that if the VA could vaccinate Veterans at their organization, it would reduce barriers, improve Veterans’ trust in the vaccine and the VA, and increase uptake:

“*It would be a lot easier if… the VA could sort of set up shop and take care of the Veterans’ vaccines right then and there. Rather than one guy goes here, one guy goes there. If that could… ever be arranged… or even for the future even with flu shots, that would be extremely beneficial. A lot of our guys are older. And they still have that [hesitation toward] going to the doctor for anything at all. And so just to see the people actually coming to them and offering them assistance, I think would build a stronger trust between the guys and the VA healthcare.” (KY GPD)*

#### Providing Educational Information

Both HPACT and GPD providers mentioned providing educational information on COVID-19 vaccines to encourage Veterans to get vaccinated. GPD organizations in MA, FL, and NJ provided information to their clients or encouraged vaccination during regular meetings, N.CA distributed educational flyers to GPD residences, and IA was creating a Q&A panel with healthcare professionals. However, the MA, IA, and FL respondents also noted that they did not want to push their own opinions onto their clients:

*“Myself and other members of our leadership team have really been focused on having conversations with our staff about kind of coaching through how to… talk openly with your clients… and maintain that rapport that takes so long to build with the population that we serve, while also not giving them misinformation or persuading them in any way.” (IA GPD)*

Conversely, the KY and S.CA GPD sites were not actively providing information as they were not seeing a lot of vaccine refusal among their clients. The S.CA respondent also noted that they were not providing educational information to Veterans because they did not believe they had the medical expertise to give advice on vaccination. Instead, Veterans were encouraged to contact their VA healthcare providers if they wanted a COVID-19 vaccine.

HPACT staff at both sites encouraged vaccination. ND and CA nurses systematically identified Veterans to target for educational outreach. Veterans were called ahead of their scheduled medical appointments to inquire about vaccination status, and if unvaccinated, were encouraged to also schedule a vaccine appointment. If Veterans had questions, they offered to answer them over the phone or have them talk to their primary care provider. HPACT providers in CA similarly noted that it was important not to push hesitant Veterans for fear that they may stop coming to clinic altogether:

*“My population…becomes very defensive. …to the point where it’s like ‘let’s just stop it, I don’t want to go there’…. I try to make it very clear that this is a topic, once they say no and once they’ve voiced their concern… for them that’s the end of it, I don’t pursue any further because what I see is that after that… their mood changes and then they’re not able to focus on their appointment and what their medical condition is… when I see…their demeanor changes then I don’t pursue too much then” (CA HPACT3)*

One CA respondent cited that the VA had provided optional trainings to teach providers how to talk about the vaccine with Veterans and mentioned that he instructed his care team on how to do “motivational interviewing” to encourage patients to get vaccinated.

Nearly all respondents agreed that Veterans need more education about the efficacy and safety of the COVID-19 vaccines. The FL GPD respondent suggested that information about how the vaccines work needs to be presented in simple visuals. N.CA GPD staff stated that messaging needs to be more targeted and culturally competent:

*“In [neighborhood], there’s this hashtag ‘can we live’. And it’s specifically aimed towards African Americans and other minorities to get vaccines and…[organizations] are putting up flyers and stuff in the community with facts and with images with people that look like them who have gotten vaccines…. [vaccine uptake] slowly started to increase. …And they also do…. these little blue packets that [GPD parent organization] drops off to our houses and the office… that they provide all of our clients that gives them updates, more facts, testimonials…” (N.CA GPD)*

#### Trusted Relationships as Sources of Information

GPD and HPACT staff at multiple sites noted that having trusted relationships with professionals sharing information with them about the vaccine was instrumental to Veterans’ vaccination decisions. The ND HPACT team continued providing face-to-face care throughout the pandemic, which they believed built trust with their Veterans. The respondents also noted that both the HPACT providers and housing program case managers discussed the COVID-19 vaccines with Veterans to ease their concerns:

*“The HPACT team, the nurses and the provider, but also… case managers… have a huge role in discussing this with their Veterans, easing any concerns and worries they may have, not mandating it, not saying you have to get this, but encouraging them, explaining how it would be helpful, and certainly in some instances know that they could utilize more services if they had been vaccinated.” (ND HPACT2)*

A CA HPACT nurse cited that primary care providers were often better able to convince Veterans to get vaccinated than nurses because of their long history with patients, and another provider noted the importance of establishing relationships with their homeless patient population. Meanwhile, GPD staff in N.CA, NJ, and MA leveraged their close, personal relationships with Veterans to listen to their concerns and persuade them to accept the vaccine:

“*I have a rapport with some of the Veterans here, and other staff with others. And we talk to them… at our monthly house meeting, the superintendent said, ‘you know myself and [interviewee], we’re vaccinated, we’re fine. It’s something you should really consider doing’.” (NJ GPD)*

Both GPD & HPACT providers noted that Veterans tend to look to their peers in making health decisions, and recommended that the VA use Veterans as messengers to encourage vaccination and overcome hesitancy:

*“I always think that the Veteran community is totally different from the regular community…I know that the military background is a very strong bond…a lot of Veterans rely on other Veterans for [advice]….so they know what they’re getting themselves into. So, I think that if a lot of Veterans have a good positive outcome with the whole vaccine, you’re going to see a lot more Veterans get it that hesitated before because they see their fellow Veterans getting the vaccine and having positive results” (MA GPD)*

## Discussion

People experiencing homelessness are particularly susceptible to severe COVID-19 disease due to underlying health conditions and are at an increased risk of exposure due to their congregate living environments [5, 6, 20]. Thus, addressing access barriers and reducing vaccine hesitancy among this vulnerable population is a public health priority for the VA [21]. The present study found that an average of 50% of Veterans receiving care and services from HPACT clinics and GPD organizations were vaccinated for COVID-19, which is consistent with findings from similar studies in the U.S. and abroad [8, 12, 22–25].

Early eligibility was important for increasing COVID-19 vaccination among people experiencing homelessness. The two GPD sites with the lowest vaccination rates (KY and IA) were in states that did not prioritize homeless shelters in their Phase 1 distribution plans [26]. GPD respondents also noted that providing easy and convenient access to the COVID-19 vaccines was key to improving uptake. Indeed, the organizations that facilitated vaccination onsite (FL and MA) had higher uptake than those that had to transport Veterans elsewhere to get vaccinated (with the exception of S.CA, whose Veteran clients seemed to be especially accepting of the vaccine). Additionally, the ND HPACT was able to get a high proportion of their Veteran patients vaccinated during vaccine blitzes that were located at the CRRC. Street and shelter-based interventions have been successful in the control and prevention of transmissible infections and increasing access to care for people experiencing homelessness [27–29]. For example, mobile clinics, pop-up clinics in convenient locations, and street outreach teams have previously been recommended to increase access to the COVID-19 vaccines [30–32]. Interagency partnerships and communication, however, will need to be strengthened for these strategies to be effective [30, 33]. Lastly, VA should consider expanding vaccine eligibility to GPD organization staff in future vaccine efforts, as they provide frontline care to Veterans in a high-risk environment.

Misinformation about the safety, efficacy, and ingredients of the COVID-19 vaccines has led to widespread reluctance among both homeless and general populations [4, 10]. Nearly all HPACT and GPD participants in this study agreed that Veterans need more information to increase confidence in the vaccines, which could include written material about COVID-19 research that is simple, clear, and culturally competent and available in a variety of languages [11, 29, 31], as well as dialogue-based education programs led by frontline professionals [29, 31, 34, 35]. Homeless service providers are particularly well-positioned to deliver health education to their clients. Yet two GPD sites in this study were not regularly offering information on the COVID-19 vaccines, with one respondent stating that they did not feel they had the expertise to give medical advice. The VA, therefore, should provide explicit guidelines for GPD staff, including trainings on motivational interviewing to encourage immunization and how to improve patient-provider relationships.

This study reinforces previous research that found high levels of cynicism toward government entities among Veterans, which in turn has led to a distrust in the COVID-19 vaccines [10, 11, 17, 34]. However, Veterans and people experiencing homelessness usually trust their healthcare providers and regard them as their most trusted source of COVID-19 vaccine information [4, 9–12]. Healthcare and housing programs provide a vital safety net for individuals experiencing homelessness during disasters [36, 37] and have been shown to improve health behaviors and outcomes [7, 29, 38, 39]. Leveraging trusted relationships between healthcare workers, homeless service providers, and their clients will likely reduce access barriers and increase COVID-19 vaccine uptake among people experiencing homelessness [8, 12, 30–32]. Rather than vaccine mandates, which may further strengthen distrust and discontent in the government and healthcare organizations [10, 29, 40, 41], incentives and social privileges (e.g., access to restaurants, sporting events, concerts) will likely be more effective at encouraging COVID-19 vaccine uptake.

Finally, Veterans have been shown to have differential health behaviors in some instances compared with the general population, likely due to past military experience, a complex relationship with the government, and military culture [10, 17, 42–45]. As recommended by respondents in this study, Veterans could be utilized as trusted advocates to provide educational outreach and encourage COVID-19 vaccination to their fellow peers. For example, the Biden Administration launched the COVID-19 Community Corps in early 2021, a nationwide network of organizations and community leaders tasked with empowering Americans to get vaccinated [46, 47]. Team Rubicon, a nonprofit organization that utilizes the skills of military Veterans to provide disaster relief, is just one of several Veteran groups within the Community Corps and has administered over 1.6 million COVID-19 vaccines across the U.S. by increasing access, building confidence, and invoking a sense of duty to protect oneself and the community at large [43, 48, 49].

*Limitations.* Because of varying data collection methods, it may not be possible to accurately compare similarities and differences between HPACT and GPD sites, nor was it the intention of this study. Since sites were nonrandomly selected and included only seven states, findings are not generalizable to all clinics and organizations serving homeless Veterans in the U.S. However, care was taken to select sites representing a diverse range of geographic areas and patient/client sizes. Lastly, GPD respondents were interviewed when the Pfizer-BioNTech vaccine was only being administered under an Emergency Use Authorization (prior to its full authorization) by the U.S. Food and Drug Administration, and before the vaccines were widely available to the public, so we were not able to assess whether these events changed processes or uptake over time. Follow-up research is recommended. Future research should also explore the possible effects of the local political environment, which was outside the scope of this study and may affect state policies dictating vaccine prioritization, vaccination and other protective mandates, or funding for resources, all of which may influence vaccination uptake.

## Conclusions

Understanding the challenges and facilitators to getting Veterans experiencing homelessness vaccinated for COVID-19 will enable the VA, healthcare professionals, and homeless service providers to develop more effective policies and educational campaigns to increase uptake among this vulnerable population. Recommendations include providing convenient access to vaccines, using trusted sources, such as homeless program staff or Veteran peers, to provide educational information about the safety and efficacy of COVID-19 vaccines, and encouraging, rather than mandating, vaccination. Lessons learned could aid entities who deliver care to people experiencing homelessness with increasing vaccine uptake for COVID-19 booster shots, seasonal influenza, and other vaccines for transmissible diseases beyond the current pandemic.

## Data Availability

The datasets generated and/or analyzed during the current study are not publicly available. They are available from the corresponding author on reasonable request, subject to approval from the ethics committee that approved the study.
